# Touch-induced pupil size reflects stimulus intensity, not subjective pleasantness

**DOI:** 10.1007/s00221-018-5404-2

**Published:** 2018-10-29

**Authors:** Roel van Hooijdonk, Sebastiaan Mathot, Evelien Schat, Hannah Spencer, Stefan van der Stigchel, H. Chris Dijkerman

**Affiliations:** 1 Experimental Psychology, Helmholtz Institute, Utrecht University, Utrecht, The Netherlands; 2 Department of Psychology, University of Groningen, Groningen, The Netherlands

**Keywords:** Somatosensory, Pupil size, Skin conductance, C-tactile

## Abstract

Interpersonal touch is known to influence human communication and emotion. An important system for interpersonal touch is the C-tactile (CT) system, which is activated by a soft stroke on hairy skin with a velocity of 1–10 cms^−1^. This system been proposed to play a unique role in hedonic valence and emotion of touch. For other sensory modalities, hedonic processing has been associated with pupil dilation. However, it is unclear whether pupil dilation can be modulated by hedonic touch. The current study investigated in two experiments how pupil size reacts to both affective and non-affective stroking. Pupil-size data were obtained to investigate differences between stroking conditions. In addition, an adjusted version of the Touch Perception Task (TPT) was used to assess subjective touch pleasantness ratings. In Experiment 1, affective (3 cms^−1^) and non-affective (0.3 and 30 cms^−1^) stroking was applied to the dorsal side of the right hand. Results revealed that stroking velocity had a significant effect on TPT-item scores, showing higher that affective touch was rated as more pleasant compared to non-affective touch, thereby replicating the previous studies. Results, however, revealed no specific pupil dilation for the 3 cms^−1^ condition; instead, a logarithmic relation was found between pupil-size dilation and stroking velocity. This relation was confirmed in a second experiment. Furthermore, the palm of the hand was used as a control site for tactile stimulation, for which similar findings were obtained as for the dorsal side of the hand. In addition, skin conductance recordings showed a pattern of response to different stroking velocities similar to pupil dilation. These results suggest that pupil-size dilation does respond to tactile input, but that this response is related to arousal caused by changes in stimulus intensity (e.g., stroking velocity) rather than specific C-tactile stimulation.

## Introduction

Pupil size is generally seen as a measure of arousal and attention and is, therefore, frequently used to index perception, language processing, memory, decision making, emotion, and even cognitive development (Mathôt and Van der Stigchel 2015; Sirois and Brisson 2014). One domain that is currently underexplored is somatosensory processing. Although the emotional and psychological effects of touch are well established (Gallace and Spence 2010), it is currently unknown how pupil size responds to innocuous touch. Given that interpersonal touch is able to evoke strong emotions in the touched person depending on the identity of the toucher and the interpersonal relationship (Hertenstein et al. 2006), one would expect to see a reflection of these effects in the pupil.

The relation between pupil size and touch has been predominantly described in pain studies. These studies have shown that the pupil dilates after various forms of painful stimulation, such as cold pressure stimulation (Tassorelli et al. 1995; Walter et al. 2005), algometer pressure stimulation (Ellermeier and Westphal 1995; Höfle et al. 2008), and electrical stimulation (Chapman et al. 1999; Vassend and Knardahl 2005). Although studies investigating the effect of innocuous touch on pupil size are scarce, other psychophysical measures of arousal have been investigated in relation to this type of touch. For example, skin-to-skin innocuous touch has been found to evoke decelerations in heart rate in humans (Gray et al. 2000; Kutner et al. 2008) and animals (Aureli et al. 1999; Lynch et al. 1974).

The arousal reducing effects of touch may be caused by activating a specific type of afferents, the C-tactile afferents (CT fibers) that have been proposed to play a unique role in processing the hedonic valence of touch (Olausson et al. 2002; Vallbo et al. 1999). CT fibers are activated by low-threshold unmyelinated mechanoreceptors that are only present in hairy skin, and project to the posterior insula, a region involved in processing of emotion and internal bodily signals (Craig 2002). Activation is triggered by soft touch with a stroking velocity range of 1–10 cms^−1^ with a peak at 3 cms^−1^, which is congruent with subjective touch pleasantness ratings showing an inverted-U pattern (Löken et al. 2009). Thus, the CT fibers are proposed to have a specialized role for the processing of hedonic, emotional, and plausibly social aspects of touch (Ellingsen 2010).

Pleasant touch is processed by a network that involves the orbitofrontal cortex (OFC) (Francis et al. 1999). The OFC is associated with assigning reward to hedonic experience (Kringelbach 2005). Interestingly, OFC activity has been found to respond to CT-fiber stimulating touch, but not to similar touch on skin lacking CT fibers (McCabe et al. 2008), providing additional support for the view that CT fibers are associated with processing hedonic valence of touch.

To our best knowledge, only one study reported the effect of innocuous tactile stimulation on pupil size. This study used affective touch, and found a larger pupil dilation for human vs machine touch, especially when touch was paired with a happy face (Ellingsen et al. 2014). Although this study suggests a relation between pupil size and touch processing, both visual and tactile stimulation were applied simultaneously, thereby limiting the ability to interpret the relation between touch and pupil size solely. Furthermore, the study only included affective touch, but no stroking velocities that do not activate CT fibers.

To investigate the relation between touch and pupil size, the current study used tactile input only, both CT optimal and suboptimal touch, and explored two characteristics of touch: stimulus intensity and subjective pleasantness. We can formulate two hypotheses. First, as stimulus intensity of painful touch has been positively related to pupil size (Ellermeier and Westphal 1995) and other autonomic responses (Kyle et al. 2009; Möltner et al. 1990), this relation is also expected for innocuous touch. Furthermore, research on other sensory domains shows that the intensity of auditory stimuli and salience of visual stimuli are also related to pupil dilation (Wang et al. 2014), suggested to be mediated via intermediate layers of the superior colliculus (Wang and Munoz 2015). Alternatively, we hypothesize that pupil size is related to subjective pleasantness of the tactile stimulus. In that case, it is expected that affective touch will cause a larger pupil dilation than non-affective touch. Hedonic processing has been associated with pupil dilation in visual (Aboyoun and Dabbs 1998; Bradley et al. 2008; Steinhauer et al. 1983) and auditory (Partala and Surakka 2003) processing, compared to stimuli that are experienced as neutral. Furthermore, the autonomic responses following painful touch increase as stimulus unpleasantness increases (Rainville et al. 1999; Tousignant-Laflamme et al. 2005), suggesting a relation between hedonic touch processing and sympathetic activation.

To explore the effects of tactile stimulus intensity and subjective pleasantness, stroking velocities that are optimal (3 cms^−1^) and suboptimal (0.3 cms^−1^, 30 cms^−1^) for targeting CT fibers are compared for their effect on pupil size. Specifically, if stimulus intensity is related to pupil size, we expect that the pupil size is largest during 30 cms^−1^ stroking, followed by 3 cms^−1^ stroking, then followed by 0.3 cms^−1^ stroking. Rather, if touch-induced pupil size reflects subjective pleasantness, we expect that pupil size in the 3 cms^−1^ stroking condition is larger than in the 30 cms^−1^ and 0.3 cms^−1^ stroking conditions.

## Experiment 1

In Experiment 1, we examined modulation of pupil size by stroking the dorsal side of the right hand. Using stroking velocities that are optimal and suboptimal for targeting CT fibers, we could explore two variables of tactile stimulation: stimulus intensity and subjective pleasantness. If stimulus intensity is related to pupil size, we expected an increase of pupil size with increasing stroking rate (30 cms^−1^ > 3 cms^−1^ > 0.3 cms^−1^). Rather, if touch-induced pupil size reflects subjective pleasantness, pupil size during 3 cms^−1^ should be larger than 0.3 cms^−1^ and 30 cms^−1^.

### Method

#### Participants

Twenty-eight subjects, with normal vision or corrected to normal vision with contact lenses, participated in this experiment (*M* = 19.14 years, SD = 1.02 years, 11 male). Participants could choose either a monetary reward or course credits for compensation. All participants gave written informed consent for participation and this experiment was performed in accordance with the declaration of Helsinki. This study was approved by the local ethical committee of the Faculty of Social Sciences of Utrecht University.

#### Apparatus and software

The right eye was recorded with the EyeTribe tracker, an infrared based eye-tracker sampling at 30 Hz. Visual stimulus consisted of a black fixation dot on a grey background, at a fixed luminance of 21.0 cd m^−2^, which was presented on a 20-in monitor (Samsung 2032BW, 1680 × 1050 px, 60 Hz). Stimulus presentation was controlled with OpenSesame (Mathôt et al. 2012).

#### Procedure and stimuli

Viewing distance (55 cm) was kept constant across participants using a chinrest. The experiment started with a nine-point eye-tracker calibration. Before each trial, a manual 1-point recalibration (“drift correction” by space bar press) was performed. In each trial, a fixation dot was presented in the centre of the screen for 18 s. Participants were instructed to keep their eyes fixated on the dot and blink as little as possible. At 3 s, a tone (440 Hz, type ‘sawtooth wave’; 92 ms decay; length 100 ms) was played as indication for the experimenter to start stroking for the remaining 15 s. Tactile stimuli were applied to the dorsal side of the right hand, using a foundation brush (goat hair; conducted pressure approx. 11.5 Pa). Stroking velocities were either optimal (3 cms^−1^) or suboptimal (0.3 cms^−1^, 30 cms^−1^) for targeting CT fibers and thereby elicited an affective experience (e.g., Löken et al. 2009; van Stralen et al. 2014). Stroking velocities for each trial were randomly assigned a priori so that each participant underwent the same trial sequence. Participants performed two blocks of 27 trials, 54 in total.

After each trial, participants rated the tactile experience with an adjusted version of the Touch Perception Task (TPT) (Guest et al. 2011). The Dutch translation of these words was used, containing eight words of the TPT with the highest proportion of variance accounted for by the factor ‘comfort’ and least covariance accounted for by the factor ‘arousal’ (Guest et al. 2011), see Table 1. Hedonic valence categorization was adopted from Ackerley et al. (2014). Ratings were assessed with a digital version of the Visual Analogue Scale (VAS-scale), handling scores in slider format ranging from 0, not at all descriptive, to 100, highly descriptive.

**Table 1:** Used TPT items with the highest proportion of variance accounted for by the factor ‘comfort’ and least covariance accounted for by the factor ‘arousal’, described by hedonic valence (as adopted from Ackerley et al. 2014)

English	Dutch	Hedonic valence description
Enjoyable comfortable	Aangenaam comfortabel	
Soothing	Geruststellend	Positive affect
Calming	Kalmerend	
Relaxing	Ontspannend	
Pleasant	Prettig	
Irritating	Irritant	Negative affect
Uncomfortable	Oncomfortabel	

### Data analysis

#### Analysis of subjective pleasantness

For the analysis of the subjective pleasantness of touch, TPT items were categorized in representing either positive or negative affect. Repeated-measures ANOVAs were conducted using SPSS 20 for both categories, to compare the effect of stroking velocity (0.3 cms^−1^, 3 cms^−1^, and 30 cms^−1^) on subjective pleasantness. Furthermore, we performed a post-hoc test for pairwise comparisons, Bonferroni corrected (*α* = 0.05).

#### Pupillometry analysis

We analyzed pupil size from stroking onset until 15 s after stroking onset. Missing pupil-size values were first linearly interpolated (given the EyeTribe’s relatively low sampling rate, this was more effective than more advanced blink-reconstruction techniques such as cubic-spline interpolation; Mathôt 2013). Next, pupil size was smoothed using a 1.7 s Hanning window. Finally, we applied subtractive baseline correction using mean pupil size during the 3 s before stroking onset as a baseline (Mathôt et al. 2018).

For each 33 ms sample separately, we conducted a linear-mixed-effects analysis using pupil size as dependent measure, stroking velocity as fixed effect, and by-participant random intercepts and slopes. Stroking velocity was log-transformed and centered on the intermediate velocity (3 cms^−1^). Effects were considered reliable when |*t*| > 2 for at least 2 s (i.e., 60 consecutive samples), approximating a criterion of *p* < 0.05.

### Results and discussion

#### TPT-item scores

**Fig. 1:**
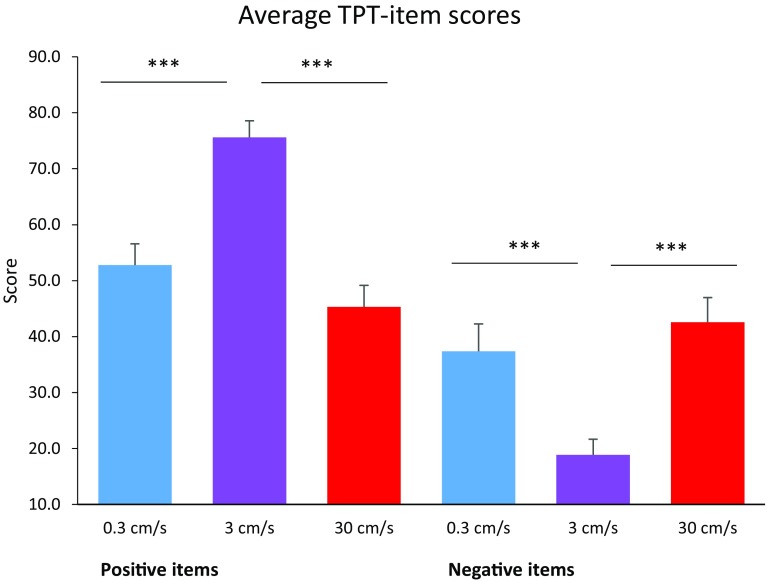
Average TPT-item scores for words representing positive and negative affect, per stroking velocity. Error bars indicate the standard error. ***p < 0.001

Repeated-measures ANOVAs revealed that stroking velocity had a significant effect on TPT-item scores representing positive affect, *F*(2, 54) = 30.191, *p* < 0.001, *η*^2^ = 0.528, and negative affect, *F*(2, 54) = 14.769, *p* < 0.001, *η*^2^ = 0.354. Positive items were rated higher, and negative items were rated lower, during 3 cms^−1^ stroking compared to both 0.3 cms^−1^ and 30 cms^−1^ stroking; *p* < 0.001 for all comparisons, Bonferroni corrected (Fig. 1).

#### Pupil size

**Fig. 2:**
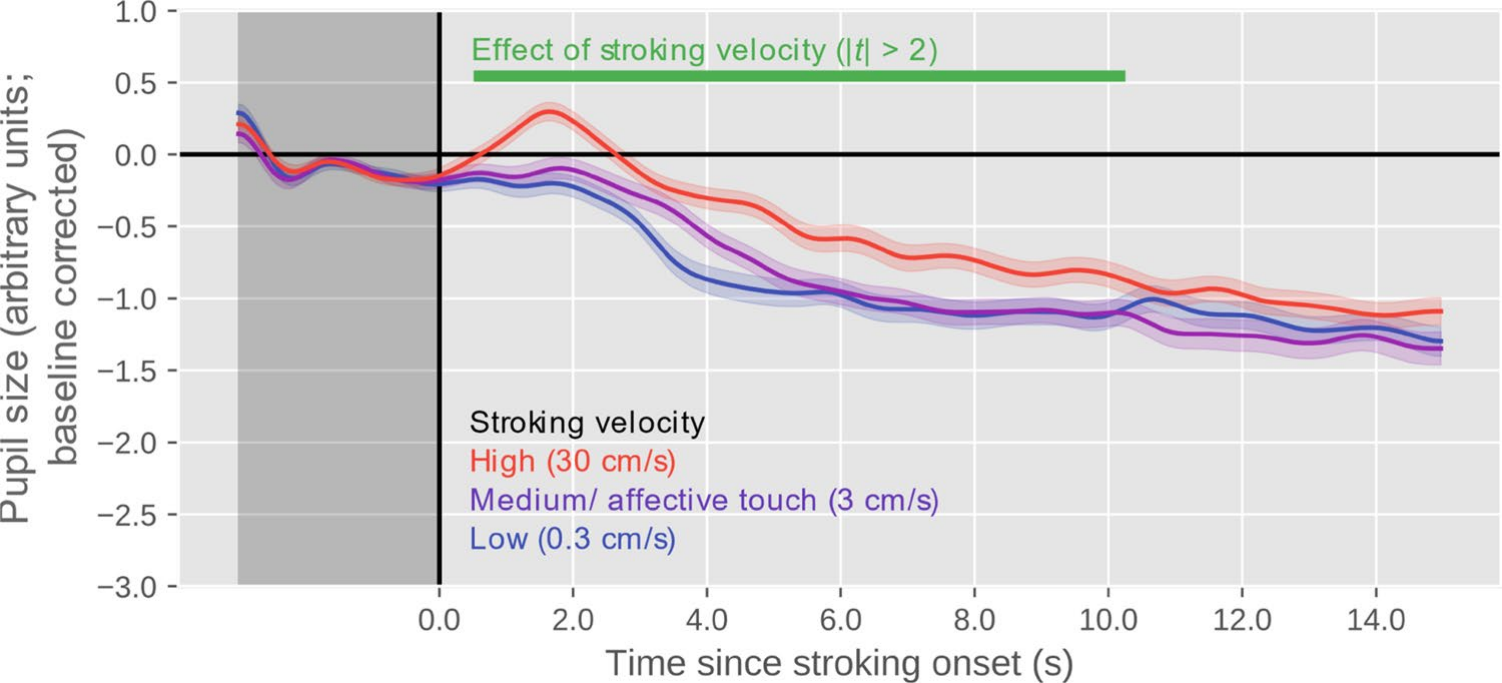
Pupil size over time, as a function of stroking velocity. Error bands indicate standard error

As shown in Fig. 2, there was a clear effect of stroking velocity, such that the pupil was larger for higher stroking velocities. There was no evidence that the pupil dilated most strongly for the intermediate (affective touch) stroking velocity.

In line with the previous research (Löken et al. 2009; van Stralen et al. 2014), in this experiment, the 3 cms^−1^ stroking was rated as more pleasant than other stroking velocities that are not known to activate CT fibers. However, pupil dilation was not specifically larger for the stroking condition rated as most pleasant. As pupil size increased with increasing stroking velocity, the results are in favour of the hypothesis that the modulation of pupil size by innocuous touch is related to the intensity of touch.

To confirm the observed pattern, a second experiment was conducted that included trials in which the ventral side of the hand was stroked. As the mechanoreceptors that activate CT fibers are only present in hairy skin (Vallbo et al. 1999), an effect of affective touch on pupil size is only expected in trials in which the dorsal side of the hand is stroked. To obtain confirmatory evidence that the effect of stroking velocity on pupil size was related to arousal, we decided to add the skin conductance response (SCR) as second measure of arousal. Since pupil size and skin conductance are both recognized as a measure for physiological arousal (Ehlers et al. 2016), it was expected that SCR follows the same pattern as pupil size, i.e., larger pupil sizes are accompanied by a higher SCR.

## Experiment 2

### Method

#### Participants

Twenty-five new subjects, with normal vision or corrected to normal vision with contact lenses, participated in this experiment (*M* = 20.11 years, SD = 1.28, 5 male). Participants received a monetary compensation for participation. All participants gave written informed consent for participation and this experiment was performed in accordance with the declaration of Helsinki. This study was approved by the local ethical committee of the Faculty of Social Sciences of Utrecht University.

#### Procedure and stimuli

In addition to the pupil-size measurements, skin conductance response (SCR) was measured during each trial. Both the dorsal (D) and ventral (V) sides of the right hand were stroked. The following sequence was repeated 15 times (90 trials in total): 3 cms^−1^ V, 30 cms^−1^ D, 0.3 cms^−1^ D, 30 cms^−1^ V, 3 cms^−1^ D, and 0.3 cms^−1^ V.

### Data analysis

#### Analysis of subjective pleasantness

The analysis of the subjective pleasantness of touch was similar to Experiment 1, where TPT items were categorized in representing either positive or negative affect. Repeated-measures ANOVAs were conducted for both categories, with stroking velocity and side (D or V) as within-subject factors. Post-hoc tests for pairwise comparisons were performed, with a Bonferroni correction (*α* = 0.05). In addition, three post-hoc paired samples *t* tests were conducted, in which we compared side for each velocity (i.e., 0.3 cms^−1^ D vs 0.3 cms^−1^ V, 3 cms^−1^ D vs 3 cms^−1^ V, etc.). We applied a Bonferroni correction, with *α* = 0.0167.

#### Pupillometry collection and analysis

We measured and analyzed pupil size in the same way as for Experiment 1, with the exception that our statistical analysis now contained an additional effect: hand side (ventral/ dorsal). Therefore, we conducted linear mixed-effects analyses (again for every 33 ms sample) using pupil size as dependent measure, stroking velocity, hand side, and the stroking velocity × hand side interaction as fixed effects, and by-participant random intercepts and slopes for all fixed effects. Hand side was dummy coded, such that − 1 corresponded to ventral and 1 to dorsal, so that the reference value (0) was the average of ventral and dorsal.

#### SCR collection and analysis

SCR recordings were made using the Biosemi ActiveTwo system (Biosemi, Amsterdam, The Netherlands) at 16 Hz sampling rate from two passive AG/AgCl electrodes attached to the palmar surface of the left middle and index finger. Saline conductor gel was used to improve signal-to-noise ratio. The ground reference point consisted of the active common mode sense (CMS) and passive driven right leg (DRL) electrode placed on the dorsal side of the left hand.

A low-pass filter of 3 Hz was applied offline to the raw SCR data to reduce interference. For each trial, − 3000 ms to + 15,000 ms response windows were selected time-locked to stroke onsets. Baseline correction was applied by subtracting the averaged SCR activity 3000 ms pre-stroke onset period from the post-stroke onset values. Data reduction was performed using Brain Vision Analyser 2 (Brainproducts, Munich, Germany). Post-stroke onset signals were entered into the same per-sample linear-mixed-effects model as we used for the pupil-size data, with the sole exception that SCR samples were taken every 62.5 ms.

### Results and discussion

#### TPT-item scores

**Fig. 3:**
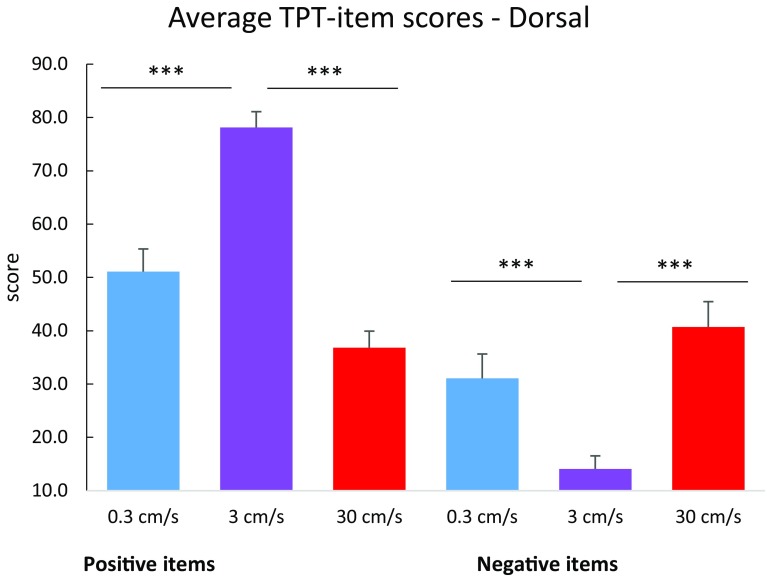
Average TPT-item scores for words representing positive and negative affect, per stroking velocity, for the dorsal condition. Error bars indicate the standard error. ***p < 0.001

**Fig. 4:**
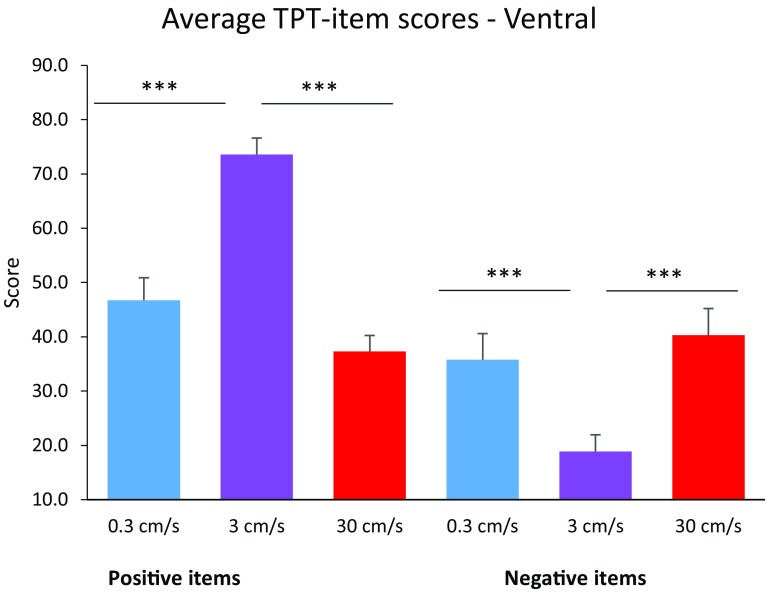
Average TPT-item scores for words representing positive and negative affect, per stroking velocity, for the ventral condition. Error bars indicate the standard error. ***p < 0.001

Repeated-measures ANOVAs revealed that stroking velocity had a significant effect on TPT-item scores representing positive affect, *F*(2, 48) = 47.325, *p* < 0.001, partial *η*^2^ = 0.664. Positive items were rated higher during 3 cms^−1^ stroking compared to both 0.3 and 30 cms^−1^ stroking, *p* < 0.001 for all comparisons, Bonferroni corrected. In addition, 0.3 cms^−1^ stroking was rated significantly more pleasant than 30 cms^−1^ stroking, *p* = 0.042. No significant effect was found for side, *F*(1, 24) = 2.465, *p* = 0.130, *η*^2^ = 0.093. An interaction effect of side × stroking velocity was found, *F*(2, 48) = 4.973, *p* = 0.011, *η*^2^ = 0.172. Post-hoc paired samples *t* tests revealed a significant effect of side for 3 cms^−1^ stroking, in which dorsal stroking was rated as more pleasant than ventral stroking; *t*(24) = 2.657, *p* = 0.014. For 0.3 and 30 cms^−1^ stroking, no effect of side was found (see Figs. 3 and 4).

For words representing negative affect, stroking velocity also had a significant effect on TPT-item scores, *F*(2, 48) = 18.266, *p* < 0.001, *η*^2^ = 0.432. Negative items were rated lower during 3 cms^−1^ stroking compared to both 0.3 cms^−1^ and 30 cms^−1^ stroking. In addition, an interaction effect of side × stroking was found, *F*(2, 48) = 3.372, *p* = 0.043, *η*^2^ = 0.123. Post-hoc paired samples *t* tests with Bonferroni correction, however, revealed no significant differences in side for each velocity. Again, no significant effect was found for side, *F*(1, 24) = 1.977, *p* = 0.173, *η*^2^ = 0.076.

#### Pupil size

**Fig. 5:**
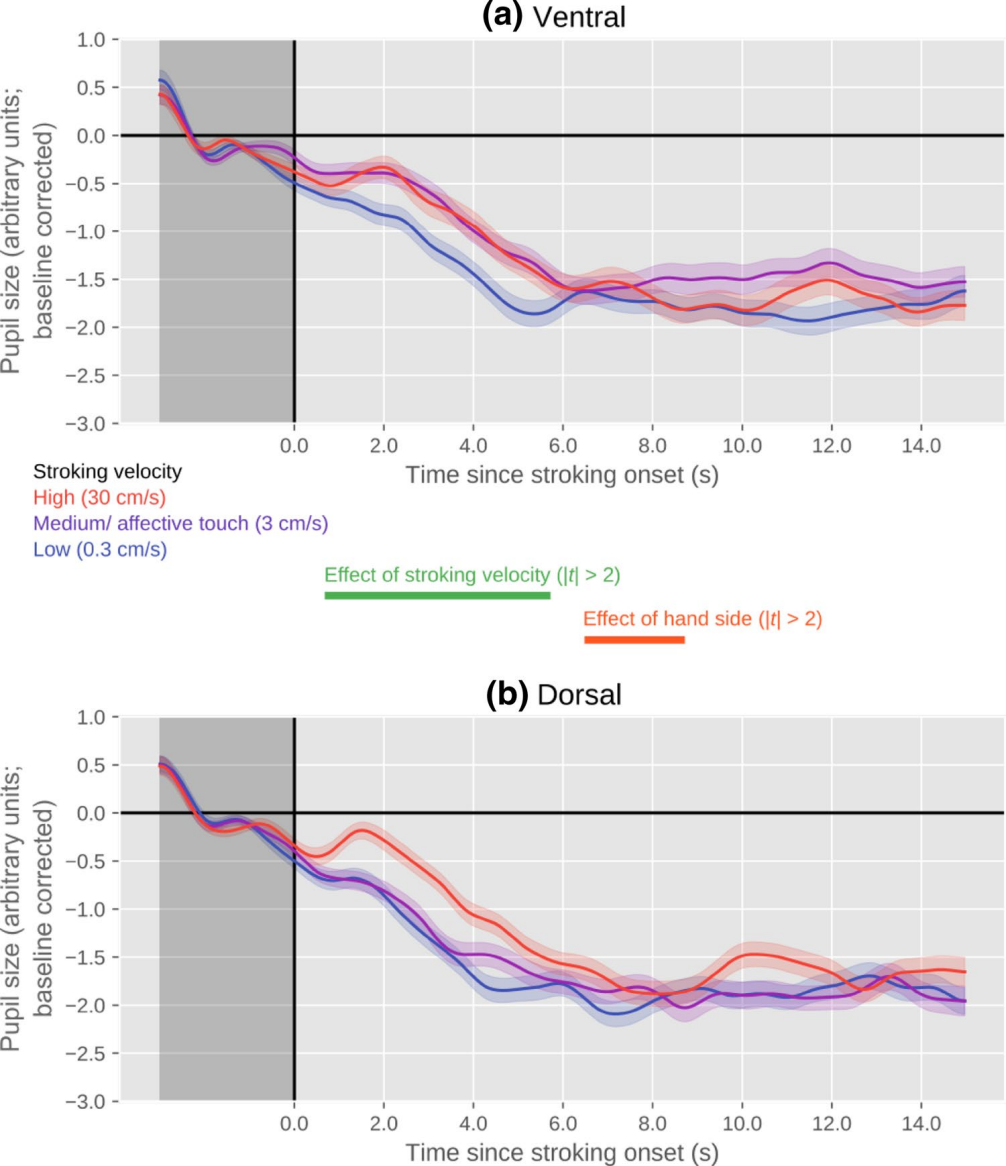
Pupil size over time, as a function of stroking velocity, separately for stroking of the ventral (**a**) and dorsal (**b**) sides of the hand. Error bands indicate standard error

As shown in Fig. 5, there was again a clear effect of stroking velocity, such that the pupil was larger for higher stroking velocities, and no evidence that the pupil dilated most strongly for the intermediate (affective touch) stroking velocity. There was also a small effect of hand side, such that the pupil was slightly larger when the ventral side of the hand was stroked. Importantly, there was no significant interaction between hand side and stroking velocity.

#### SCR

**Fig. 6:**
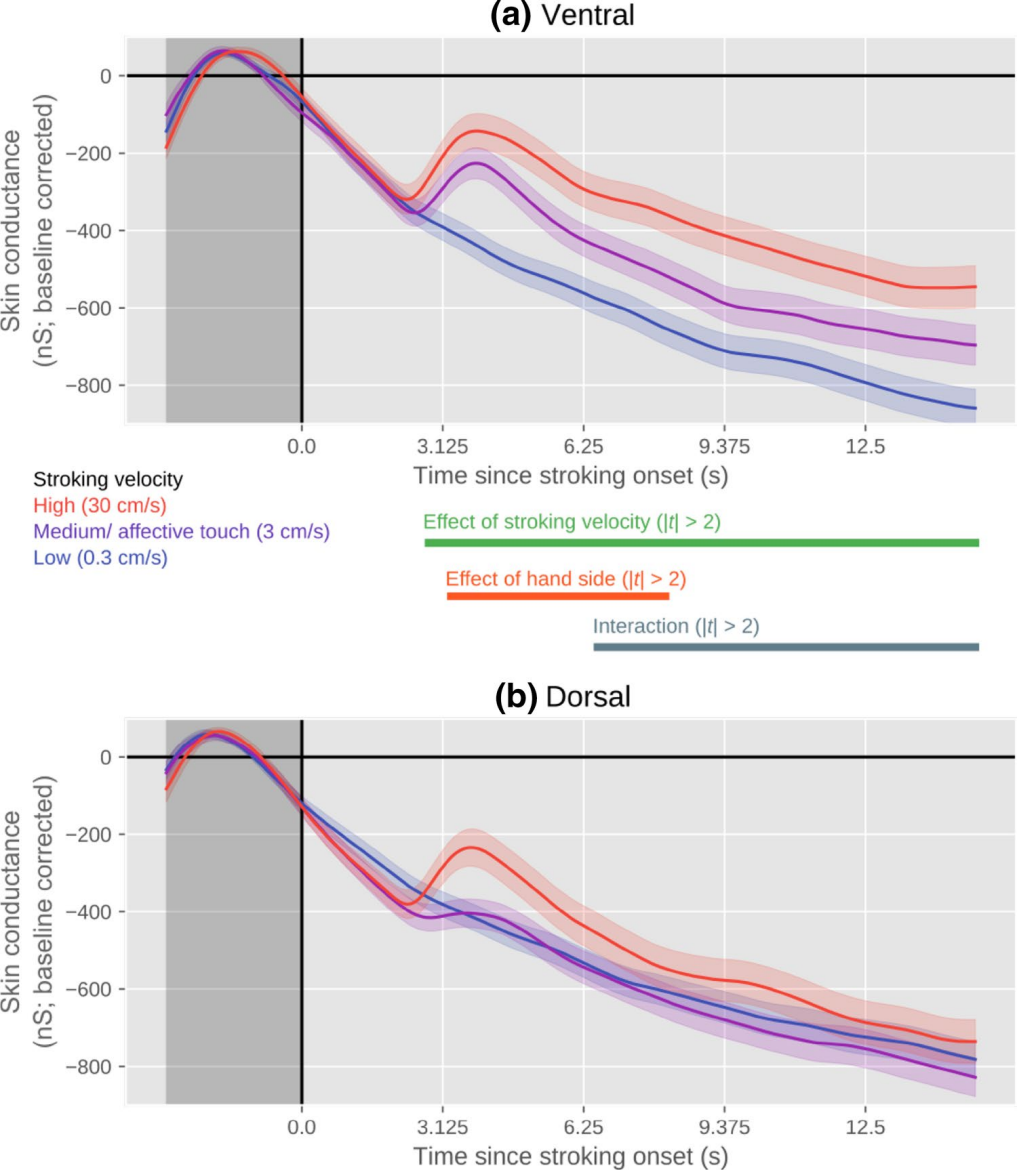
Skin conductance over time, as a function of stroking velocity, separately for stroking of the ventral (**a**) and dorsal (**b**) sides of the hand. Error bands indicate standard error

As shown in Fig. 6, the SCR results largely resemble the pupil-size results. There was a clear effect of stroking velocity, such that skin conductance was higher for higher stroking velocities, and no evidence that the pupil dilated most strongly for the intermediate (affective touch) stroking velocity. There was also an effect of hand side, such that skin conductance was higher when the ventral side of the hand was stroked. Finally, there was a stroking velocity × hand side interaction, such that the effect of stroking velocity was most pronounced when the ventral side of the hand was stroked.

## Discussion

Overall, the results of experiment 2 confirmed those of Experiment 1. Analysis of the subjective pleasantness of touch revealed that words representing positive affect were rated higher, and words representing negative affect were rated lower, during 3 cms^−1^ stroking compared to both 0.3 and 30 cms^−1^ stroking. In addition, 3 cms^−1^ stroking was rated significantly more positive during dorsal compared to ventral stroking. This implies a specific affective touch effect, caused by stimulation of CT fibers.

With respect to the pupil-size data, for both dorsal and ventral stroking, results of the mixed-effects model revealed a significant effect of stroking velocity on pupil size.

SCR results for ventral stroking are in line with the pupil-size data, showing a clear logarithmic relation between stroking velocity and SCR. For dorsal stroking, 3 cms^−1^ and 0.3 cms^−1^ did not significantly differ in SCR. One possible explanation is that this lack of an effect is a consequence of the same order in which the stroking velocities were presented. Inspection of the data shows that the overall signal for the 0.3 cms^−1^ stroking was uplifted by the baseline correction. A lower baseline SCR prior to 0.3 cms^−1^ dorsal stroking may be explained by an expectancy effect: a 30 cms^−1^ dorsal trial was always followed by a 0.3 cms^−1^ dorsal trial, which, therefore, could be predicted by the participant resulting in a lower arousal.

All results combined, these findings are congruent with the hypothesis that stimulus intensity of touch, rather than hedonic processing, is the main cause of an increase in sympathetic activation.

## General discussion

The present study explored the effect of innocuous stroking on the human hand on alterations in pupil size. The overall pattern of results is congruent with the hypothesis that pupil size reflects stimulus intensity, not subjective pleasantness. In the first experiment, 3 cms^−1^ stroking was rated as more pleasant than other stroking velocities that are known not to activate CT fibers. If pupil size reflects subjective pleasantness, rather than stimulus intensity, larger pupil sizes were expected to be accompanied by higher pleasantness ratings, which was not the case. In fact, a robust effect of stroking velocity on pupil size was found, where a constant increase in stroking velocity was accompanied by a constant increase in pupil size.

To further explore any possible effects of affective touch on sympathetic activation, a second experiment was conducted including stroking on the ventral side (i.e., the palm of the hand which is not innervated by CT fibers) and skin conductance responses (SCR). Regarding subjective pleasantness, we found that 3 cms^−1^ dorsal stroking was rated as more pleasant than 3 cms^−1^ ventral stroking. On both the dorsal and ventral sides, 3 cms^−1^ stroking was rated as most pleasant with no differences between 0.3 cms^−1^ and 30 cms^−1^ stroking, similar to the first experiment. In addition, in line with the first experiment, an effect of stroking velocity on pupil size was found, in which a constant increase in stroking velocity was accompanied by a constant increase in pupil size. This effect was present in both dorsal and ventral conditions. SCR results of the dorsal condition showed no significant difference between 0.3 cms^−1^ and 3 cms^−1^ stroking. SCR results for ventral stroking showed the same logarithmic pattern as seen in the pupil-size data. Thus, the effect of affective touch was marginal and had no clear influence on sympathetic activation.

Although there was no clear evidence of affective touch-induced sympathetic activation (as measured by the pupil size and skin conductance) in the current study, another study suggests that CT-fiber stimulation might still result in sympathetic activation, although much smaller than induced by simultaneously stimulating the A-beta fibers. Olausson et al. (2007) tested two subjects who lacked A-beta fibers, but had a functional CT system. Their results showed that soft stroking, that activated the CT fibers, can induce sympathetic skin responses. However, the findings of the current study suggest that any sympathetic response that affective touch may induce is normally overshadowed by the sympathetic response as a consequence of increasing the amount of A-beta tactile input.

Besides the somatosensory domain, a relation between stimulus intensity and pupil dilation has also been argued for visual and auditory processing. Furthermore, it has been found that micro-stimulation of the superior colliculus evokes a pupil response that is comparable to visual or auditory evoked pupil responses (Wang et al. 2014). Interestingly, in this study by Wang et al. (2014), responses to audiovisual stimuli were well predicted by a linear summation of each modality response. Regarding the present study, it would be interesting to compare and combine somatosensory with auditory and visual input in a similar design, to test if the superior colliculus is a modality-independent coordinator of saliency-induced pupil responses.

To conclude, in this study, we observed effects of innocuous stroking on the human hand on alterations in pupil size. In the previous studies, stimulus intensity of painful touch has been positively related to pupil size (Ellermeier and Westphal 1995) and other autonomic responses (Kyle et al. 2009; Möltner et al. 1990). Combined with our findings, we pose that the relation between stimulus intensity and sympathetic activation is not restricted to painful touch, but that this relation can be applied to touch in general. Measures of stimulus intensity other than stroking velocity, such as pressure or vibration frequency, however, remain to be tested.
